# Integration of Sentinel-1 and Sentinel-2 Data for Land Cover Mapping Using W-Net

**DOI:** 10.3390/s20102969

**Published:** 2020-05-24

**Authors:** Massimiliano Gargiulo, Domenico A. G. Dell’Aglio, Antonio Iodice, Daniele Riccio, Giuseppe Ruello

**Affiliations:** Department of Electrical Engineering and Information Technology (DIETI), University Federico II, 80125 Naples, Italy; domenicoantoniogiuseppe.dellaglio@unina.it (D.A.G.D.); antonio.iodice@unina.it (A.I.); daniele.riccio@unina.it (D.R.); giuseppe.ruello@unina.it (G.R.)

**Keywords:** synthetic aperture radar, image segmentation, rice monitoring, convolutional neural network, data fusion, time-series analysis, sentinel data

## Abstract

In this paper, we present a new approach to the fusion of Sentinel 1 (S1) and Sentinel 2 (S2) data for land cover mapping. The proposed solution aims at improving methods based on Sentinel 2 data, that are unusable in case of cloud cover. This goal is achieved by using S1 data to generate S2-like segmentation maps to be used to integrate S2 acquisitions forbidden by cloud cover. In particular, we propose for the first time in remote sensing a multi-temporal W-Net approach for the segmentation of Interferometric Wide swath mode (IW) Sentinel-1 data collected along ascending/descending orbit to discriminate rice, water, and bare soil. The quantitative assessment of segmentation accuracy shows an improvement of 0.18 and 0.25 in terms of accuracy and F1-score by applying the proposed multi-temporal procedure with respect to the previous single-date approach. Advantages and disadvantages of the proposed W-Net based solution have been tested in the National Park of Albufera, Valencia, and we show a performance gain in terms of the classical metrics used in segmentation tasks and the computational time.

## 1. Introduction

Global World Monitoring is nowadays supported by a huge number of satellites [1] providing precious data in several applications, such as agriculture, biodiversity, and hydrology [2]. Remote sensing images can be continuously utilized to monitor land cover/land use changes around the world with extremely accurate precision. Multispectral/Optical images, such as Sentinel-2 or Landsat TM, are mainly employed in applicative scenarios thanks to their capacity to represent natural color images of wide portions of the Earth surface, providing information about vegetation, risk management (floods, forest fires, and so on), water monitoring, and much more [3,4,5,6]. In addition, a plethora of indices, such as the Normalized Difference Vegetation Index (NDVI) [7,8,9], the Normalized Difference Water Index (NDWI) [10,11,12], and others have been developed to provide quantitative estimates of selected surface covers [6]. However, multispectral sensors are unusable in presence of cloud cover and this prevents the possibility to guarantee a continuous monitoring. In order to overcome this drawback, recent works [13,14,15,16] proposed the use of Synthetic Aperture Radar (SAR) imaging sensors, able to acquire even in presence of clouds. In literature, the polarimetric SAR (PolSAR) [17,18], the polarimetric SAR interferometry (PolInSAR) [19], and the Multi-chromatic analysis PolInSAR (MCA-PolInSAR) [20] methods have been used to extrapolate information about the physical proprieties of targets. Furthermore, the bottleneck of using single sensor is an inherent trade-off between spatial and temporal resolution. For instance, in many works information from satellites with daily basis global information and coarser spatial resolution is utilized (MODIS: 250 m; Sentinel-5: 8 km, NOAA - AVHRR: 1 km), but even the data from satellites with finer spatial resolution and coarser temporal resolution are equally exploited and useful (i.e., Landsat TM: 16 days; SPOT/HRV: 26 days) [21,22,23,24,25]. In this context, in order to obtain a pixel-wise land cover classification with a dense revisit time, the fusion of the features extracted by both the multi-spectral and SAR data is approached. In fact, in the last decades a plethora of works used different data fusion techniques to combine specific bands and polarizations of optical and SAR sensors, respectively [26,27,28]. In this scenario, the ESA Copernicus program is developing Sentinel missions, specifically for RS applications, aiming to ensure a continuity of data, never seen before, with the huge number of launched satellites. Thus, the Sentinel-1 and Sentinel-2 missions approach these problems by providing data with higher revisit time than the other satellites at finer spatial resolution, and higher spatial resolution than the daily basis sensors. Some information about these two missions is reported in Table 1.

**Figure 1:**
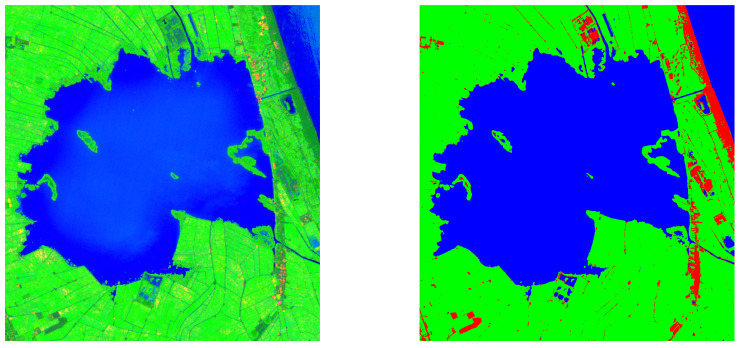
The false RGB colour of the lake under investigation (R: VH polarisation, G: NDVI, B: MNDWI), on the left, and the segmentation maps (R: Bare soil, G: Vegetation, B: Water), on the right. The latitude and the longitude of the images go from N 39.372 to N 39.288, and from W −0.407 to W −0.304, respectively. Both in a specific date: 24 August 2019.

In particular, in the last decade an increasing interest in Deep Learning (DL) has incentivized the use of the methods based on the Convolutional Neural Networks in remote sensing (RS) data fusion. In a rising number of works, the data fusion is approached by using the Deep Learning (DL) architectures because of their effectiveness and appeal in computer vision and accordingly in RS context [26,29,30]. These approaches are very helpful in global monitoring for a plethora of applications. In literature the image segmentation is addressed using different approaches: (i) superpixel segmentation methods [31,32], (ii) watershed segmentation methods [33,34], (iii) level set segmentation methods [35,36], and (iv) deep learning segmentation methods [37,38]. In this work, we used a deep learning method benefiting from the accuracy of the segmentation maps obtained with Sentinel 2 data and the temporal capacity of Sentinel 1, as in our previous work [38]. Specifically, we investigate the use of the Sentinel-1 data (Table 2) in the Natural Park of Valencia, Albufera, in Spain, to monitor the presence of water, rice or bare soil. The rice growing dynamics have regularly to be monitored and can take advantages of: (i) the high accuracy of land cover mapping, (ii) an improved spatial resolution of the remote sensing data, and (iii) even better revisit frequency from the RS products. A portion of the area under investigation and the data on stage are represented in the Figure 1. The paper is organized as follows. In Section 2 we present the site under investigation, and describe the S1 and S2 data used in this work. In Section 3 we explain the proposed W-shaped Network (briefly W-Net, hereafter) architecture. In Section 4, the implementation of the training phase and the results are shown in details. In Section 5 we discuss the performances of this branch of approaches, in terms of accuracy, time, memory occupation, and so forth. The concluding session is devoted to summarize the main findings and to draw future perspectives of this work.

## 2. Materials

### 2.1. Site Description

**Figure 2:**
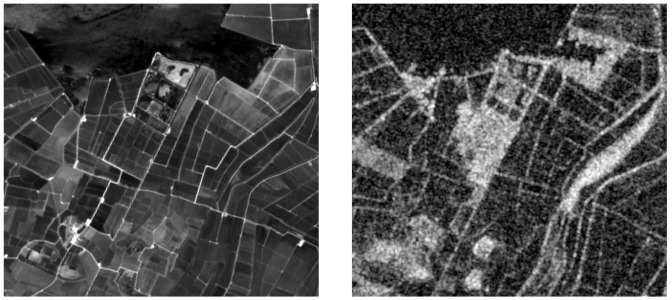
Visual comparison between Sentinel 2, B2 band on the left, and Sentinel 1, VV polarization on the right.

The study area shown in Figure 1 is a wetland area located in the Natural Park of Albufera, in the vicinity of Valencia, Spain. In Figure 1 we used the World Geodetic System (WGS84) ellipsoid as geographic reference system; latitude and longitude coordinates are reported in the image caption. The Albufera region is a wetland with a rich biodiversity, where rice farmers, fishermen, ecologists, fishes and migratory birds share the access to water. In this context, water volume and quality are affected not only by seasonality and climatic conditions, but also by human activities. In particular rice farmers pump or transfer water in accordance with their cultivation needs. This complex space- and time-variant environment constitutes an excellent benchmark for land cover mapping research. In addition, this area is particularly interesting from the applicative point of view, because many stakeholders are trying to use remote sensing tools for water management issues with an increasing need of technical algorithms to improve the usability of the data. In this area due to the rapid vegetation dynamics the fusion of SAR and optical data became very useful. In fact, the importance of combining these sources is highly helpful when cirrus and cloud presence reduces the usability of data from passive sensor. In this work we used S1 and S2 data, whose main characteristics are reported in the next two Section 2.2 and Section 2.3. The information from these sensors comes from different interaction between electromagnetic fields and the considered scene. In particular, in Figure 2 we highlight the difference between these two products, and focus on the speckle noise, that it is noticed in the S1 image, but not in S2 bands. This speckle effect is the consequence of the small objects presence (at the wavelength scale) and of the coherent nature of illumination in the resolution cell. Due to the microscopic details of these objects’ shapes and positions the signals may interfere constructively or destructively. Thus, the overall effect is a “salt-and-pepper” noise on the S1 images.

### 2.2. Sentinel-2

The twin Sentinel-2 satellites provide global coverage with a five days revisit time at the equator using 13 bands spanning from the visible to the short-wave infrared (SWIR) and showing different spatial and spectral resolutions, as reported in Table 3. Sentinel-2 data are provided with different levels of processing. Level-1C (L1C) is provided in Top Of Atmosphere (TOA) reflectances, and Level-2A (L2A) is provided in Bottom Of Atmosphere (BOA) reflectance images, obtained from the parameters of the L1C.

The Sentinel-2 images allow obtaining rich information about vegetation, water, and so on. The most common land monitoring applications are based on temporal series of indices, for instance the Normalized Difference Vegetation Index (NDVI) [7], the Normalized Difference Water Index (NDWI) [10], and much more. The NDVI and the NDWI are defined as in [39]:

(1) $$NDWI=\frac{\rho_{8}-\rho_{11}}{\rho_{8}+\rho_{11}}$$

where $\rho_{8}$ is the Near Infrared (NIR) band, centered at the wavelength of 842 nm; while $\rho_{11}$ is the SWIR band, centered at 1610 nm, and

(2) $$NDVI=\frac{\rho_{8}-\rho_{4}}{\rho_{8}+\rho_{4}}$$

where $\rho_{8}$ is the above-mentioned NIR band, and $\rho_{4}$ is the Red band, centered at 665 nm.

In this work, we use the above-mentioned indices to provide a segmentation map by means of a simple threshold of the NDWI and NDVI. In particular, the threshold is set to zero for both indices. From this indices-based approach, we only defined 3 classes using the mentioned thresholds: (i) *water*, when the values of NDWI are higher than zero, (ii) *vegetation* for values of NDVI higher than zero, and (iii) *bare soil* for values lower than zero both in NDVI and NDWI.

### 2.3. Sentinel-1

The European Space Agency (ESA) launched the satellite Sentinel-1A, the first of a constellation composed by two twin radar sensors, in April 2014. The two Sentinel-1 satellites provide C-band images in both singular and dual polarization with a repeat cycle of 6 days. These data are freely available, enabling a number of Earth monitoring applications [13,16]. S1 can acquire images in four acquisition modes as Stripmap (SM), Interferometric Wide Swath (IW), Extra Wide Swath (EW) and Wave (WV) with different processing levels. The specifications of the data used in this work are given in Table 2. In the following analysis, the images were generated from the high-resolution Level-1 Ground Range Detected (GRDH) product with a 10-m pixel size. The Sentinel-1 GRDH Level-1 data are pre-processed using the official ESA SentiNel Application Platform (SNAP) software before training phase. In particular data calibration, and terrain correction with a 25-m Shuttle Radar Topography Mission (SRTM) data has been performed. After this processing, we obtain the calibrated backscattering coefficient $\sigma_{0}$. The sigma nought $\sigma_{0}$ is a normalized dimensionless measure for the intensity of a pixel, and depends significantly on polarisation, incidence angle, and wavelength. We provide $\sigma_{0}$ for both VV and VH polarisations.

#### Sentinel-1 Analysis

**Figure 3:**
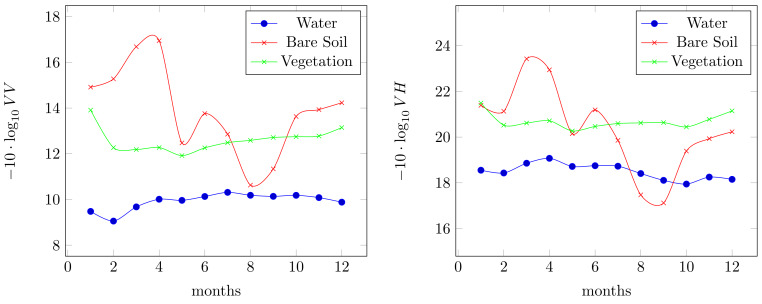
Multi-temporal information about the considered VV and VH polarisations, month by month, where with VV and VH we denote the $\sigma_{0}$ of these two polarisations.

We focus our data analysis on the entire 2019 year. The considered dataset obtained by twin Sentinel-1 satellites (details in Table 2) includes 12 acquisitions, one for each month. In the same time interval 12 S2 acquisitions have been acquired. In Figure 3 we summarize the temporal profiles per VV and VH polarizations of the considered classes: bare soil, water and rice. As expected the water and vegetation classes are more easily distinguishable, instead the bare soil class is more challenging. In Figure 3 it is equally noticed that the bare soil class is distinctive in winter seasonal condition, and in summer assumes values that can be easily misclassified with other two considered classes. The information provided by VH is similar in terms of multi-temporal trend and different in terms of the range of intensity values. The combination of these two polarizations are surely effective in the segmentation context. Observing the multi-temporal trends, from the combination of these two polarisations, we can see that some specific configurations allow us to distinguish one class from the others. For instance, the water class is separable from the vegetation using dual polarization information, since the $\sigma_{0}$ in VV and VH for vegetation is lower than for water. Thus, in Figure 3, we even found the vegetation trend in the top of the water, because we showed $-10\cdot log_{10}\left(\cdot \right)$. But, the bare soil class is more misleading because its average range of values is wider both for the VV and VH polarisations.

## 3. Proposed Method

**Figure 4:**
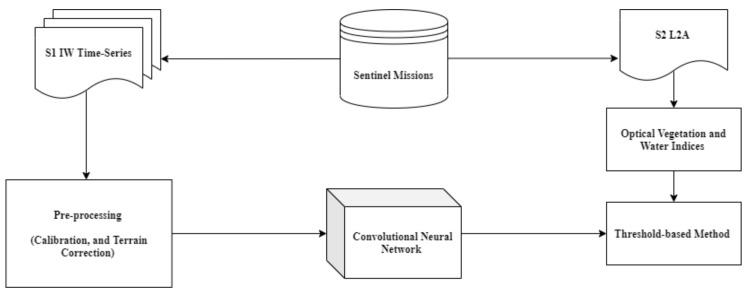
The general workflow.

Our proposed method is based on a supervised deep learning algorithm, whose general workflow is drawn in Figure 4. The core of the considered workflow is the Convolutional Neural Network that is fed with four combinations of VV and VH polarisations as input stack, as reported in Table 4. As output, we consider the above-described S2 segmentation map, obtained by threshold-based technique. Compared with the previous work [38], the novelties introduced in this paper are:

- a novel deep learning architecture, called W-Net because of its W-shaped structure;
- the deep learning data fusion approach to the case of multi-temporal data;
- and a different segmentation map as reference, obtained by using the L2A product. In the previous work, we used a segmentation map, provided by the L2A product that included a huge number of invalid pixels.

**Figure 5:**
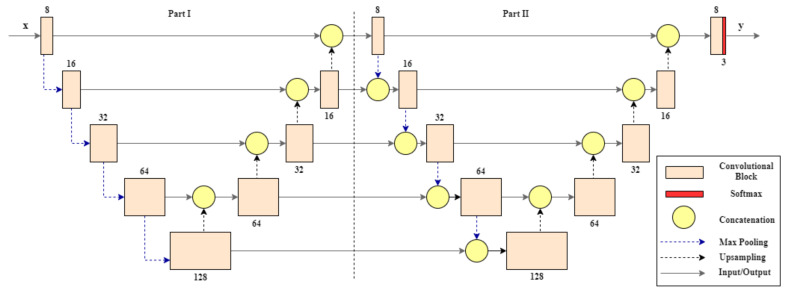
The proposed W-Net architecture.

In the multi-temporal input configuration, we consider three dates: one is the closest to the target date, and the others are the next closest dates, before and after the target date. In addition, the S2 data are reprojected into the geographical raster of the S1 ones to properly set up the training phase. It consists of the resampling of one product (the slave) into the geographical raster of the other (the master). In our case, the master and the slave products are, respectively, the S1 and the S2 data. The main innovation of this work is in the use of a deep learning architecture, called W-Net. These CNN-based solutions are commonly used in a lot of remote sensing applications [40,41,42]. However, this specific architecture is used in a remote sensing context for the first time in this work. The structure is drawn in Figure 5. The W-Net, designed as an evolution of the U-Net architecture [43], is inspired by [44,45]. The W-shaped design is identical to [44], but the two networks differ in the interconnection between the two twin U-Nets and as well as in the application point of view. In fact, in our case, this design is performed in supervised training to have a large number of convolutional levels, keeping a low number of trainable parameters given the presence of max pooling, while in [44] this design is considered to perform unsupervised training. The overall architecture is composed of two U-Net structures in series. Each U-Net structure is organized in two paths: a contracting and an expansive one as reported in [46]. The first contracting part is an encoder, composed by a sequence of blocks. The blocks have as basic component the batch normalization layers, and 3×3 convolutional layers interleaved by rectified linear unit (ReLU). This basic component is considered twice to obtain a single convolutional block, as reported in Figure 5. The blocks are connected each other by the max pooling 2×2 layers. The max pooling is useful to preserve the main target information and to reduce the number of parameters. For instance, after the max pooling a 2×2 matrix corresponds to a single value, obtained as the maximum of the considered matrix. Furthermore, each max pooling layer corresponds doubling in the number of feature maps. We limited the number of the features because this limitation gives a better compromise between processing time and accuracy of results. In fact, the number of kernels for any convolutional layers increases from 8 to 128 in the contracting path, and goes back up to 8 in the expansive path. The second expansive path is the decoder part. It is composed by convolutional and upsampling layers. The downsampling of the first part and the upsampling of the second one is performed four times, and so the last feature maps obtained by the upsampling path have the same size of the input images.

The first part, that has the same structure of the U-Net, performs a concatenation of the feature maps from the encoder path to the corresponding feature maps from the decoder part, and this concatenation is very helpful in recovering information lost during the convolutional and max pooling operations. After the last concatenation achieved by the first U-Net-like part, there is another sub-architecture that again has a U-Net structure. This second part is equal to the previous, but the output of the max pooling layers is concatenated with the output of the blocks at the same level in the previous U-Net part. All these concatenations fed the basic encoder blocks of this second part of the W-Net. In the second U-Net, after the last concatenation between the first block of the encoder path and the upsampling of the last block of the decoder path, there is an additional block equal to all others. But, the last layer is a 1×1 convolutional layer that matches the number of feature maps to the desired number of classes, and uses a softmax activation function. This W-Net based segmentation approach requires a supervised learning, and so we have to realize input-target (x,y) samples to begin the training phase. In fact, we consider the S1 SAR data with different combinations of VV and VH polarisations as input, and the indices-based segmentation maps from the S2-L2A product as target. The (x,y) samples are used to train the model. In more details we use a objective function based on the Intersection over Union (IoU) to be more effectual in error back-propagation [47]. The IoU function can be defined as:

(3) $$IoU=\frac{I}{U}=\frac{y\cap \hat{y}}{y\cup \hat{y}}$$

where I and U are Intersection and Union, respectively, and *y* is the reference map and $\hat{y}$ is the predicted one. The IoU can be also expressed in terms of True Positive (TP), False Positive(FP), and False Negative (FN):

(4) $$\frac{I}{U}=\frac{TP}{\left(TP+FP+FN\right)}.$$

Specifically, the IoU loss is computed on the objective function by averaging over the mini-batches samples at each updating step of the learning process:

(5) $$L_{IoU}=1-IoU=1-\frac{1}{N}\sum_{n=1}^{N}\frac{y_{n}\cap \hat{y_{n}}}{y_{n}\cup \hat{y_{n}}}$$

where *N* is the batch size in training phase, $y_{n}$ is the *n*-th target and $\hat{y_{n}}$ is the *n*-th predicted map, dependent on all the parameters of the network. Then, we consider this IoU loss into the objective function, defined as in the following form:

(6) $$argmin_{w}=1-IoU$$

where w are the weights of the convolutional layers. Further, we adopt the Adam optimiser implementation of Tensorflow, and we consider a 0.02 learning rate, and the other parameters are configured as in [48]. In networks with a large number of convolutional layers a good initialization of the weights is crucial. In fact, we start the training by the Glorot inizialization [49]. Despite the scarsity of data for training phase we have verified that this random inizialization of the network weights gives considerable results. In particular, we consider four input configurations (see Table 4) that differ each others in the composition of the input stack x, while the output y is always the classification provided by the L2A product of Sentinel-2 at the target date.

## 4. Experimental Results

In this section, we first report a brief description of the evaluation metrics used in the classification tasks and the comparative methods (Section 4.1). Then, we provide numerical and visual results (Section 4.2).

### 4.1. Classification Metrics

**Figure 6:**
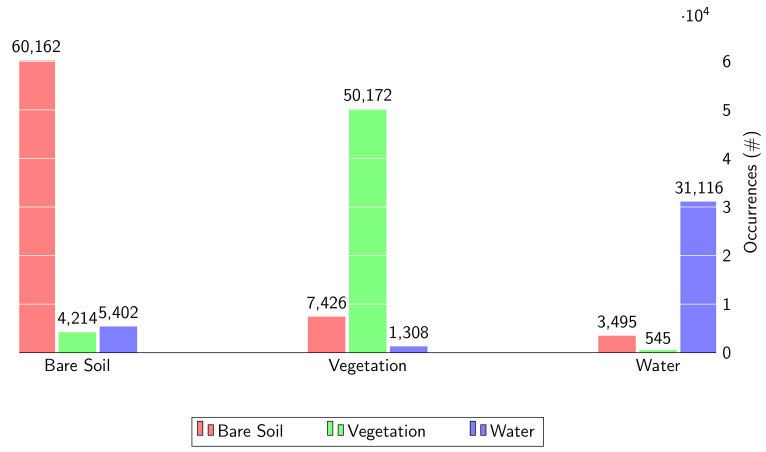
Bar plot of the Confusion Matrix.

To assess our model, we use a set of metrics derived from the confusion matrix. The confusion matrix allows us to give a complete evaluation of the performance, as shown in Figure 6. In fact, all wrong and correct predicted values are reported in this table. Thus, the main metrics used in classification evaluation are accuracy, precision, recall, and F1-Score. These metrics are defined as follows:

(7) $$Accuracy=\frac{TP+TN}{TP+FP+FN+TN}$$

(8) $$Precision=\frac{TP}{TP+FP}$$

(9) $$Recall=\frac{TP}{TP+FN}$$

(10) $$F1=\frac{2\cdot Recall\cdot Precision}{Recall+Precision}$$

where TP, TN, FP and FN are numbers of true positives, true negatives, false positives, and false negatives, respectively. Ideally, FP and FN should be equal to zero, and consequently, accuracy, precision, recall and F1 score would be equal to 1. These metrics require reference and, likewise for the training data, we have produced the target using the indices-based technique from the Sentinel-2 L2A product. However, this indices-based classification is affected by errors, that constitute a limitations in training phase. Of course in order to further improve the performance of the deep learning approaches the target must be more accurate.

### 4.2. Compared Methods

In this section, we compare the proposed architecture with others, commonly used in segmentation task: shallowNet [50], SegNet [51], LinkNet [52], U-Net [43], and FPN [53]. To understand the main differences between all these state-of-the-art(SoA) solutions, we start from the first part of the W-Net that corresponds to the U-Net solution, as mentioned before. But, in this comparative analysis the U-Net has the double of kernels in every convolutional layers. Then, the LinkNet has the same architecture of U-Net, but the concatenation layers are replaced by sum operator. In U-Net and LinkNet solutions, the disadvantages with respect to the proposed architecture are the lower number of convolutional levels, and the huge number of parameters. But the number of parameters in some cases could be an advantage, because it is possible to detect more complex link between input and output. Besides, the SegNet is characterized by the same encoder-decoder structure, but every block of the decoder path upsamples its input using the transferred pool indices from its corresponding block of the encoder path. This approach highly loses spatial information, with a limited number of parameters. The FPN and the shallowNet correspond to a different basic architecture. In fact the shallowNet is a cascade of four convolutional layers interleaved by ReLu activation function, and the FPN is an architecture based on a parallel analysis of the input at different scales. This capability of the FPN allows to simultaneously obtain a segmentation at different scales. In Table 5 we show the number of learnable parameters and memory occupation of the state-of-the-art (SoA) considered architectures. Specifically, all architectures use the max pooling strategy, except for the shallowNet that is the lightest CNN with the lowest number of parameters. ShallowNet can be helpful in presence of small datasets, as in this case. However, in the specific context, the other solutions are trained starting from pre-trained weights, and so they obtained better results than the shallowNet. All SoA deep learning solutions are trained on all input stack configurations, and are adapted on the different dimensions of the input stacks. Some SoA competitors have a lot of parameters, and this amount of learnable parameters allow to obtain appreciable results, and give an idea of the goodness of these deep learning methods, confirmed by the results obtained by the proposed W-Net. In fact our method improves the performance in terms of all metrics, in particular in the whole configuration with the three dates in the input stack.

### 4.3. Numerical and Visual Results

In order to build a dataset with different rice growing phase we collected twelve dates for training, one for each month. This dataset is more general than the data used in our previous work [38], and gives more information about the site under investigation. In fact, in the above-mentioned work only 5 dates were used to train SoA architectures. Even in this case we only left apart ten 128×128 patches for testing (each around the lake of the Natural Park, and from different dates), 128×128 patches for training were selected from all dates in the remaining segments. Overall, 10,000 patches were collected and randomly grouped in mini batches composed by 32 patches for the implementation of the Adam-based training. Furthermore other 1000 patches were also selected for the validation analysis. The training phase was performed for just 5 epochs. The average assessments over the test images are gathered in Table 6. This table provides objective evaluation of the results, and in fact we can see that the proposed solution outperforms all compared SoA solutions on all indicators. Instead, in Table 7 we report the same analysis in terms of all metrics, but in the bottom of the table (last four rows) it is possible to understand the importance of the dual polarization and multi-temporal approach in input stack. In fact, the third (dual polarization) configuration gives better results in terms of accuracy and F1-score than the single ones. On the other hand, the dual polarization configuration (III in Table 7) shows a highly worse precision compared to the configuration I, the VH polarization configuration.

**Figure 7:**
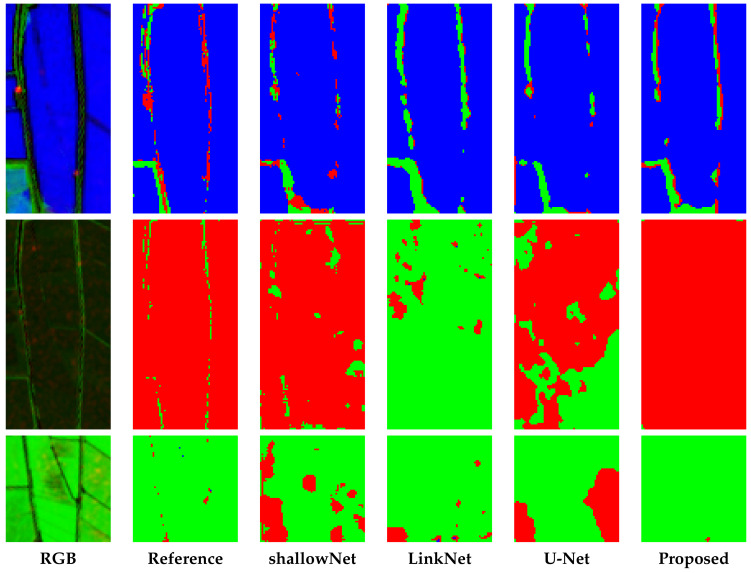
Zoomed details of segmentation results for a subset of SoA approaches, and our proposed method (W-Net). In the first column, a false colour images (R: VH, G: NDVI, B: MNDWI) is shown; in the other columns, the segmentation maps obtained with the methods under comparison are depicted. In all the segmentation maps green, red and blue pixels represent Vegetation, Bare Soil and Water, respectively.

Observing the Equation (8), it is clear that this technique overestimates the false positive. Further, in this bottom part of the Table 7, it is also possible to underline the importance of the multi-temporal configuration. In fact, the fourth configuration outperforms all others. In order to perform a complete numerical evaluation we consider the confusion matrix (Figure 6) related to the prediction of the proposed method. From this figure, we deeply observe the capability of the W-Net to distinguish considered classes, and in particular a good capability to separate vegetation from water. Infact we notice that the pixels of the vegetation class correspond to the water value in 2.22% of cases, and the water pixels to the vegetation ones in 1.50%. Instead, according to Section 2.3, the bare soil class is more difficult to classify, but the shown results for this class seem to be as accurate as the ones for the other classes. In fact, the pixels of the bare soil class do not correspond to the right value, in 13.11% of cases. Moreover, the vegetation pixels are wrongly recognized as bare soil in 12.61% of cases, and the water pixels in 9.94%. Let us now look at some samples from the test images. Samples in Figure 7 show some details used for test, associated with different rice growing conditions. From this visual inspection we compare some results of the SoA approaches, and the proposed one. The visual analysis allows us to understand the results of the proposed methods. In fact, in the first row we can see that our proposed W-Net correctly consider a large part of the pixels with respect to the others, that wrongly detect the red pixels. In all these shown details, as in the second and in the third rows, we can see that the small lines are totally lost, but the errors are lower than in other solutions. Moreover, as we can see in Figure 7, the problem is also related to not accurate reference.

## 5. Discussion

In this section, we explore in more details some results underlining the importance of using multi-date input stack and a lighter architecture to ensure good performances and preserve the time consuming aspect.

### 5.1. Single Date and Multi Date

**Figure 8:**
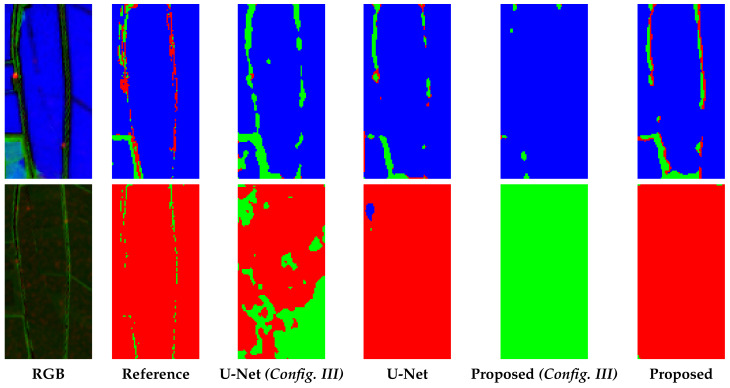
Zoomed details of segmentation results. In the first column, a false colour images (R: VH, G: NDVI, B: MNDWI) is shown; in the other columns, the segmentation maps obtained with the methods under comparison are depicted. In all the segmentation maps green, red and blue pixels represent Vegetation, Bare Soil and Water, respectively. With (Config. III) we consider the dual polarization configuration both for the U-Net and the proposed W-Net.

In Table 6 we have seen that the proposed architecture outperforms all others, and in Table 7 we underline the improvement in multi date configuration. In particular, we focus on U-Net and the proposed W-Net. In multi-temporal configuration we can assume that the convolutions of the considered architectures mitigate the effect of the multiplicative speckle noise. Furthermore, each considered competitor has a consistent improvement in multi date configuration with respect to the single date ones. This gain in terms of all metrics is really remarked in W-Net, and this is because in W-Net we have much more convolution layers, and a lower number of parameters than in the U-Net. Thus, we assume that more levels of convolution in W-Net than in U-Net correspond to a better multi-temporal despeckling effect. Taking into account that in the Sentinel-1 pre-processing we did not consider the speckle filter, we obtain a good segmentation maps with a reduction in terms of computational complexity. This is underlined by the W-Net that increases its performances more than the U-Net in multi date configuration without increasing too much the training times. In fact the W-Net solution highly improves the performance (+0.2 for accuracy, +0.14 for precision, +0.28 for recall, and +0.29 for F1-score) accepting an increase in training time of just 8.6 s per epoch, instead the U-Net has a lower increase in performance with a considerable increase in training times (+21.6 s per epoch). Since all the simulations are perfomed on Graphics card NVIDIA GeForce GTX 1050 and the training phase is carried out for just 5 epochs, the total training time is equal to 448 s (just over 7 min) and 1046 s (just over 17 min), respectively. For further validation, we consider the visual inspection (in Figure 8), and in particular we can notice that in the first row of Figure 8 the multi-temporal configuration is able to identify the linear paths visible in target image and in the false colour RGB. Especially in the W-Net these bare soil linear paths are not present in the single date configuration. Furthermore, in both networks with the multi-date configuration we are able to better classify these bare soil pixels that in the single date U-Net solution are incorrectly recognized as vegetation (V) and in the W-Net as water (B). In the second row of Figure 8 we can see that in particular the W-Net improves the classification reducing the misclassified pixels, instead the multi-data U-Net in this specific case obtains a worse classification than the single-date one. Thus, the use of deeper input stacks improves the accuracy of segmentation maps. Furthermore, in order to provide a complete comparison and analysis about the performances, we repeated the training phase 20 times for these two architectures using different initial random weights. From these simulations, we concluded that the proposed method achieves greater robustness and repeatability than the U-Net. In fact, the standard deviations of Accuracy, F1, Precision and Recall turn out to be 0.009, 0.013, 0.011, and 0.012, respectively, for the W-Net, and 0.081, 0.061, 0.053, 0.076, respectively, for the U-Net.

### 5.2. Computation Time, Number of Parameters and Memory Occupation

In this section, the results are compared in terms of architecture complexity, and in terms of computation time. The proposed W-Net has the better trade-off between computational complexity, memory occupation, and performances. In fact this proposed architecture is the second for memory occupation, time, and is largely better than all other networks in terms of all metrics used in this paper to evaluate the goodness of the obtained segmentation maps. The shallowNet has the lowest number of parameters, and consequently the lowest time per epoch, and memory occupation, however its performances are worse than the more deeper solutions. On one hand, the proposed architecture is extremely deep, but is composed of a small number of parameters. This feature gives noticeable advantages. On the other hand this became the main disadvantage of the proposed method in presence of greater datasets. In fact greater datasets require more parameters to avoid the overfitting problem.

## 6. Conclusions

In this study, a novel approach for data fusion image segmentation has been proposed and validated over the Albufera Lagoon, in Valencia (Spain). Experimental results indicate that the proposed architecture outperforms the SoA deep learning networks, and in particular we underline the improvement in multi-date configuration. In particular, the use of multi-temporal input stack gives better results considering some well-known segmentation metrics: accuracy, precision, recall, and F1-score. In this multi-temporal configuration we can assume that the convolutional layers of the considered networks mitigate the effect of the multiplicative speckle noise, and it is helpful in time management, because of lower training time required for deeper input stacks. In particular, the proposed W-Net achieves the better trade-off between processing time, memory occupation, and performances. The outputs of our proposed architecture encourage us to use this information in multi-temporal rice growing analysis, in particular to densify the multi-temporal analysis obtained by the use of the S2 data. This solution opens the way to further innovations. In particular, new investigations could focus on integrating the proposed data fusion with data acquired by different sensors to improve the segmentation performances, because it is possible to benefit from a higher revisit time considering more sensors. In addition, we can achieve more robust results considering more complex architecture in W-Net framework. However, this requires an increase in computational complexity and the creation of largest dataset for training. Eventually, the obtained results allow us to investigate the Sentinel-1 data in order to train other architectures, for instance a novel architecture based on recurrent neural networks that are helpful in multi-temporal analysis as shown in [54].

## Figures and Tables

**Table 1:** Summary of the used satellite sensors. The * symbol indicates that we only considered 6 days revisit time around the considered S2 date.

Data	Type	Satellite	Spatial Resolution	# Images	Minimum Revisit Time	Considered Revisit Time	Polarization Bands
Satellite Images	Synthetic Aperture Radar (SAR)	S-1	10 m	36	6 days	6 days *	VV + VH
	Multi-Spectral	S-2	10 m	12	5 days	1 month	$\rho_{8}$, $\rho_{11}$, $\rho_{4}$

**Table 2:** General information about the considered SAR data.

Specifications	Sentinel-1A Data
Acquisition orbit	Descending
Imaging mode	IW
Imaging frequency	C-band (5.4 GHz)
Polarization	VV, VH
Data Product	Level-1 GRDH
Resolution Mode	10-m

**Table 3:** The 13 Sentinel-2 bands.

Spatial Resolution [m]	Spectral Bands (Bands Number)	Wavelength Range [μm]
10	Blue (2), Green (3), Red (4), and NIR (8)	0.490–0.842
20	Vegetation Red Edge (5,6,7, 8A) and SWIR (11,12)	0.705–2.190
60	Coastal Aerosol (1), Water Vapour (9), and SWIR (10)	0.443–1.375

**Table 4:** Different input stack considered in training phase.

Configurations	No. Input Bands	Description	Considered Times
I	1	$VH_{i}$	1
II	1	$VV_{i}$	1
III	2	$VV_{i},VH_{i}$	1
IV	6	$VV_{i},VH_{i}$	0, 1, 2

**Table 5:** General information about the SoA architectures.

Models	# Parameters	Time per Epoch [s]	Memory
ShallowNet	45.6 k	32.0	191 k
SegNet	1.8 M	63.2	7.17 M
FPN	6.9 M	711.0	26.8 M
LinkNet	4.1 M	428.8	30.9 M
U-Net	8 M	209.2	30.9 M
Proposed	1.2 M	89.6	4.75 M

**Table 6:** Numerical Results for all architectures in the whole configuration, that means three dates and dual polarisation.

Methods	Metrics			
	Accuracy	Precision	Recall	F1
ShallowNet	0.8271	0.7743	0.7651	0.7639
SegNet	0.8240	0.7691	0.7610	0.7596
FPN	0.8418	0.8107	0.7746	0.7801
LinkNet	0.8310	0.8083	0.7623	0.7667
U-Net	0.8846	0.8567	0.8318	0.8405
Proposed	**0.9121**	**0.8860**	**0.8682**	**0.8762**

**Table 7:** Comparison between single polarization, single date and multi date in terms of all metrics.

Methods	Configuration	Metrics				Time per Epoch
		Accuracy	Precision	Recall	F1	
U-Net	III	0.7938	0.7503	0.7002	0.6812	**177.6**
U-Net	IV	**0.8846**	**0.8567**	**0.8318**	**0.8405**	209.2
Proposed	I	0.7162	0.7306	0.6091	0.5832	70.2
Proposed	II	0.7263	0.6865	0.6280	0.5806	**63.6**
Proposed	III	0.735	0.6563	0.6299	0.6213	81.0
Proposed	IV	**0.9121**	**0.8860**	**0.8682**	**0.8762**	89.6
